# Are monkeys sensitive to informativeness: An experimental study with baboons (*Papio papio*)

**DOI:** 10.1371/journal.pone.0270502

**Published:** 2022-07-05

**Authors:** Anne Reboul, Olivier Mascaro, Nicolas Claidière, Joël Fagot

**Affiliations:** 1 Aix Marseille Univ, CNRS, LPC, Marseille, France; 2 Station de Primatologie-Celphedia, CNRS UAR846, Rousset, France; 3 Institute for Language, Communication and the Brain, Université Aix-Marseille, CNRS, Aix-en-Provence, France; 4 Université de Paris, CNRS, Integrative Neuroscience and Cognition Center, Paris, France; Texas A&M University, UNITED STATES

## Abstract

Informativeness (defined as reduction of uncertainty) is central in human communication. In the present study, we investigate baboons’ sensitivity to informativeness by manipulating the informativity of a cue relative to a response display and by allowing participants to anticipate their answers or to wait for a revealed answer (with variable delays). Our hypotheses were that anticipations would increase with informativity, while response times to revealed trials would decrease with informativity. These predictions were verified in Experiment 1. In Experiments 2 and 3, we manipulated rewards (rewarding anticipation responses at 70% only) to see whether reward tracking alone could account for the results in Experiment 1. We observed that the link between anticipations and informativeness disappeared, but not the link between informativeness and decreased RTs for revealed trials. Additionally, in all three experiments, the number of correct answers in revealed trials with fast reaction times (< 250ms) increased with informativeness. We conclude that baboons are sensitive to informativeness as an ecologically sound means to tracking reward.

## Introduction

Human linguistic communication recruits an array of cognitive abilities. While many of them are not specific to language, they are nonetheless crucially involved in linguistic production and linguistic interpretation. Investigating which of these abilities are present in other species, and notably in other primate species is central to any approach to language evolution. The target of the present study is informativeness, a central factor in human linguistic communication. A signal A is more informative than another signal B if A reduces uncertainty more than does B. To take a simple example, suppose you are faced with an array of mugs, some of which are yellow and only one is yellow with a chipped handle. Your friend Paul asks you to pass him his mug. Given that you don’t know which mug it is, his demand will be more informative if he says “Please, pass me my mug: it’s the yellow one with a chipped handle”, rather than if he says “Please, pass me my mug: it’s the yellow one”.

The concept of informativeness is central to human linguistic communication because it is an important factor in interpreting utterances beyond their literal meaning, a process known as *pragmatic enrichment*. To take a simple example, if you ask who came yesterday and the answer is “Paul came”, you will interpret it as meaning that *only* Paul (and no one else) came. Yet, the utterance, in itself, does not exclude that other people, among a set of people who could have come (e.g., Paul, Peter, and Mary), came. The process through which the strengthened or enriched interpretation is reached is called *exhaustification*. It corresponds to the exclusion of alternatives more informative than the utterance used. Given that the utterance was not “Peter, Paul, and Mary came” (or “Peter and Paul came” or “Paul and Mary came”), the possibility that Paul was accompanied by either Peter and Mary or both of them is excluded. Hence the utterance “Paul came” can be interpreted as “*Only* Paul came”. Exhaustification thus supposes a sensitivity to degrees of informativeness. To exclude the more informative alternatives, the addressee must rank the different alternatives on a scale of informativeness. Exhaustivity interpretations are rife in human linguistic communication (and in human communication in general), including such constructions as focus, cleft-sentences, and scalar implicatures, among others (for an overview, see [1]). Such mechanisms are coherent with the view that interpretation is guided by assumptions about the cognitive utility of the communicated information, where cognitive utility is cashed out in terms of cognitive cost (how difficult it is to reach the interpretation) and cognitive benefit (how informative the interpretation is, when it has been accessed). While Sperber and Wilson [2] have formulated this idea explicitly, it was already outlined in Grice [3], and has been developed and formalized more recently by Frank [4] and Degen and Tanenhaus [5], among others. An important point is that while some of these theoretical approaches to interpretation appeal to higher level processes, involving mind reading about the communicator’s intentions (e.g., [3]), most others are perfectly compatible with more or less automatic, relatively low-level processes, involving no form of conscious reasoning.

The process of exaustification might be used by some non-human primate species as well. Arnold et al. [6] noted that for Campbell’s monkeys in the Taï forest (where there are eagles and leopards), *krak* is interpreted as referring to leopards, while on the Island of Tiwaï (where there are eagles but no leopards), it is interpreted as signaling a general danger. Schlenker et al. [7] suggested that *krak* might have the same “lexical” meaning in both locations (a general alarm call), but that in Taï, it triggers a process of exhaustification, enriching its meaning to refer to leopards. Based on the description of Campbell’s monkey alarm calls given in [6, 8, 9], Schlenker et al. [7] have identified as the relevant set of alternative alarm calls <*krak*> and <*hok*>, where *krak* signals the presence of danger *and hok* signals the presence of an eagle. In that case, *hok* is strictly more informative than *krak* (it is appropriate in a subset of the situations in which *krak* is appropriate). According to Schlenker et al., when *krak* is used, it is interpreted as non-*hok*, which leads in Taï (where there are leopards) to a leopard interpretation, while on Tiwaï (which is leopard free), it is interpreted “literally” as referring to a general danger. This proposal supposes that Campbell’s monkeys are sensitive to informativeness, a reasonable assumption, given the ecological usefulness of such an ability, not only in communication, but in decision making in general. Indeed, sensitivity to informativeness might be widespread among nonhuman primates, a hypothesis that the present study investigates, targeting Guinea baboons (*Papio papio*). In recent years, baboons have been shown to have abilities directly relevant to language acquisition and evolution in humans, such as a similar vocal system, associative learning, statistical learning, long-term memory and cultural evolution (for an overview, see [10]). This makes them a good choice for any study relevant to language evolution.

Informativeness has been investigated in young children (see [11]), showing that infants and toddlers use informativeness to guide their search behaviors and are able to represent for themselves the specific piece of information that they lack. They also use informativeness in communicative contexts, notably when inferring the meaning of a new word and adapting their own communicative behaviors to be as informative as possible to their addressees (e.g., [12, 13]). On the other hand, to our knowledge, there has been no investigation of sensitivity to informativeness in monkeys. However, there is an important literature on metacognition in nonhuman primates and metacognition, while different from sensitivity to informativeness, has links with it.

Metacognitive processes monitor and control the operations of the primary cognitive processes. Monitoring expresses itself in confidence judgments regarding one’s own knowledge, while control produces strategies to learn what one does not know. Both informativeness and metacognition have to do with uncertainty: Confidence is higher when uncertainty is low and vice versa. Moreover, choosing learning strategies aims at reducing uncertainty and thus should select those with the most informative potential. Thus, the experimental investigation of metacognition is of some relevance to that of informativeness.

What is remarkable in the metacognition literature is the robustness of the results throughout the different studies. First, in studies that had only monkeys as participants (usually macaques, see [14–17]), the monkeys showed appropriate use of the Uncertainty response (UR) as well as transfer to new tasks and generalization. Second, in all studies comparing humans and monkeys [18–23], the results were very similar between the two groups. There were strong inter-individual differences in both monkeys and humans. Both groups had a suboptimal use of the UR (used it less than they should have to maximize the rewards). Finally, and most importantly, both groups nevertheless made appropriate use of the UR. While these similarities do not imply that nonhuman primates enjoy the full, conscious abilities for metacognitive reasoning that humans do, they clearly mean that they have some metacognitive abilities (for a full discussion, see [24]), both relative to monitoring and to control. This suggests that sensitivity to informativeness might also be found in nonhuman primate species. The main goal of the current research was to assess baboons’ sensitivity to informativeness.

## Experiment 1

### Materials and methods

#### Participants and living conditions

Participants were 24 Guinea baboons (*Papio papio*) belonging to the CNRS Primate Centre in Rousset-sur-Arc (France), median age 10 years, min = 2, max = 24; 7 males). The baboons belonged to two social groups, one comprising two adult males and three adult females and the second six males and thirteen females. The larger group was housed in group 700m2 outdoor enclosure connected to two 24m2 trailers containing the test systems and a 16m2 indoor enclosure. The smaller group was housed in a 24m2 outdoor enclosure connected to an 18m2 trailer containing the test systems and a 9m2 indoor enclosure. Outdoor enclosures contained various kinds of objects for behavioral enrichments, such as climbing structures or stones of various sizes that the baboons can manipulate. The indoor enclosure also contained platforms for resting at night. Baboons received their daily food ratio at 5 pm (fruits, vegetables, and monkey chows), and water was provided ad libitum within each enclosure. All baboons were familiar with the matching procedure, due to previous testing.

### Animal welfare

Our research used automated learning devices which have been described in detail in Fagot & Bonté, (2010) [25]. This procedure uses an automated radio-frequency identification of the subjects within each test system, making it possible to test the individuals without removing them from their social group. Use of this procedure is known to reduce the stress level of the monkeys during the experiment, as demonstrated by reduced cortisol levels and a reduced number of behavioral stereotypies (Fagot et al. 2014) [26]. Daily observations of the baboons by ethologists and the animal care staff of the CNRS primatology station guarantied animal welfare during the experiment. Any sign of discomfort, injury of sickness would have triggered an health check by the animal care staff, but none of the baboons showed such signs during the research. Our experiment involved no anesthesia, analgesia, or euthanasia. All our subjects stayed in their home social groups during the research, and remained in their home group after completion of the experiment.

### Apparatus

The two outdoor enclosures where the baboons lived were connected to a total of 14 (n = 10 for the large group, n = 4 for the smaller group) Automated Learning devices for monkeys (ALDM) which have been described in detail in [25]. Each ALDM comprised (1) a RFID reader for the identification of the subject, (2) a 19-inch touch screen for stimulus presentation and the recording of the behavioral responses, and (3) a food dispenser delivering a drop of dry wheat within the ALDM cubicle when a correct response is made. The baboons were implanted with two biocompatible 1.2 by 0.2 cm RFID microchips injected into each forearm and were automatically identified by the test system whenever they entered an ALDM test chamber. Thanks to a network server connecting all ALDMs, the identity of the subject served to resume the trial list at the place at which the subject left it at its previous visit in the same or a different ALDM. In short, this system allowed independent test regimen for each baboon who was maintained in its social group, in absence of capture, and the baboons were tested at their own pace irrespective of ALDM they decided to use. The experiment was controlled by a software testing program written by JF using E-prime (Version 2.0 professional, Psychology Software Tools, Pittsburgh, PA, USA). The voluntary participation of the subjects reduces stress levels, as inferred from the significant decrease in salivary cortisol levels as well as the frequency of stereotypies [26].

### Test procedure

#### General test procedure

**Fig 1:**
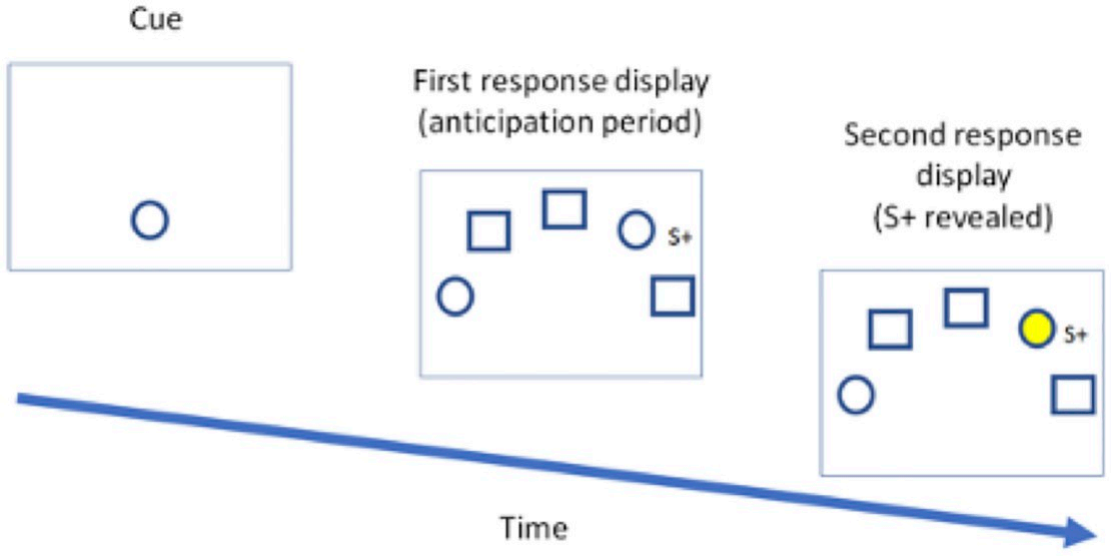
Experimental display.

Our research used an experimental paradigm which is illustrated in Fig 1. At the onset of the trials, the baboon perceived a cue stimulus which was presented at the bottom of the screen. It then had to touch the cue to trigger the presentation of the second display corresponding to the anticipation period. Depending on the test condition, the anticipation display contained either 1, 2, 3, or 4 duplications of the cue along with respectively 4, 3, 2 or 1 replications of the distractor, for a total of five stimuli on the screen. When the display contained more than one instance of the cue stimulus, one of them was randomly assigned as the S+. In other words, among the stimuli matching the cue, only one, the S+, was reinforced. The other matching stimuli were not reinforced. By contrast, when only one instance of the cue was displayed, S+ corresponded to that unique stimulus. The task for the subject was to find S+ and to touch it on the screen. Informativeness in this procedure is defined relative to the number of possible choices: the more matching stimuli there are on the response screen, the less informative the cue, and vice versa. As the likelihood of finding S+ decreased with the number of cue repetitions, the informativeness of the cue decreased accordingly. Thus, the informativeness of the cue is in inverse ratio to the number of matching stimuli: displays with 4-3-2-1 matching stimuli means that the informativeness of the cue will be rated 1-2-3-4 respectively. A correct selection of S+ during the anticipation display triggered the delivery of a food reward and finished the trial. An incorrect choice (i.e., the baboon either choose a duplication of the cue other than S+ or a distractor) during the anticipation period gave rise to a 3 sec time-out indicating that an error has been made. However, the subject had also the option of not responding during the anticipation period. In that case, the anticipation display was replaced after a variable delay (see below) by another response display similar to the previous one, with the only difference that S+ was now revealed by a yellow colour. Touching S+ provided a food reward at that stage and ended the trial. Touching another stimulus (even if it matched the cue) was considered an error which triggered a 3 sec- time out with a green screen. All error trials (whether anticipated or not) were followed by a new randomly generated trial. A maximum of 5 sec was allowed to touch the screen on the first (cue display) or third (revealed) display. A failure to touch the screen during these delays aborted the trial. Aborted trials were not recorded and were represented again at the next identification of the subject. All stimuli measured 180X180 pixels. They were presented on a 760X1024 pixels black background. All the data and metadata for the experiments can be found at https://osf.io/uqxsz/.

#### Training procedure

This procedure pursued two different goals. First, we wanted to have the baboons discovering that they had to match the stimuli considering their identity. Second, we wanted to have the baboons discovering that they could produce anticipation responses during the anticipation display. Training proceeded in 6 consecutive steps each involving blocks of 100-trials comprising 65 matching trials randomly intermixed with 35 revealed trials. Each block was repeated in a random order until the baboon reached 80% correct, reinforced responses within a block, for both matching and revealed trials. Training in block 1 only involved three items in the response display. In matching trials, they were the S+, identical to the cue, and two distractors different from the match. In revealed trials, the three response stimuli were all different from the cue (and hence there was no match present in the display), and S+ turned into a yellow color after an anticipation period of 100ms. Training in blocks 2 to 6 followed the same procedure as in block 1, except that the anticipation period was expanded to 200, 300, 500, 700 and 1000ms respectively.

#### Test phases

Three consecutive test phases were proposed once the subjects reached the learning criterion in training block 6 (80% of correct, reinforced responses). In test phase 1, the subjects were exposed to 30 repetitions of 40-trial test blocks, which were only composed of randomly ordered trials. Trials within a block involved the presentation of the cue stimulus which was either a circle (50% of the trials) or a square (50%), followed by the response display containing 5 response items made up with either 1, 2, 3 or 4 repetitions of the cue in addition to 4, 3, 2 or 1 repetition of the distractor. The distractors were squares when the cue was a circle, or *vice versa*. In test phase 1, the duration of the anticipation period was set to 1000ms. Test phases 2 and 3 used the exact same procedure as for test phase 1, except that the duration of the anticipation period was increased from 1000ms to 1500ms (test phase 2) and 2000ms (test phase 3), respectively. We expected that the lengthening of the anticipation period would enhance the number of anticipation responses.

#### Ethics statement

This research on baboons was carried out in accordance with French and EU standards and received approval from the French Ministère de l’Education Nationale et de la Recherche (approval # APAFIS-2717-2015111708173794-V3). Procedures used in Experiment 1 and the following were also consistent with the guidelines of the Association for the Study of Animal Behavior.

### Statistical analysis

The aim of our analysis was to determine whether baboons could use the informativeness of the cue to respond to the test display. We reasoned that if that were the case, then we could (1) expect the number of anticipation trials to increase with informativeness and/or (2) see shorter reaction times for informative cues for revealed trials. Anticipations were coded as a binary variable (1 if there was anticipation, 0 if not) for each trial.

When analyzing revealed trials, we found a bi-modal distribution of reaction times with a first small peak at around 150ms and a second one around 500ms (see S1 Fig in S1 File). We reasoned that the first peak reflected fast responses that were prepared by the baboons in anticipation of the signal and that their number should increase with informativeness. Therefore, we decided to analyze separately fast responses (RT < 250ms) from other slower responses.

We ran mixed-effects regression models using the lme4 package developed in R [27, 28] and calculated p-values using lmerTest [29]. All models contained a fixed effect of Informativeness and random effects to control for the non-independence of repeated measures with intercepts for Subjects as well as by-Subject slopes for the effect of Delay when several delays were present. We used negative binomial models when the dependent variable was the number of anticipations and gaussian models when analyzing reaction times. Non-parametric tests were used to analyze fast responses, given their small number.

### Results

Fourteen monkeys passed training and completed the full set of test trials (median age 10 years, min = 4, max = 22; 3 males).

### Do monkeys use informativeness to anticipate their response?

#### Increase in the number of anticipations

**Fig 2:**
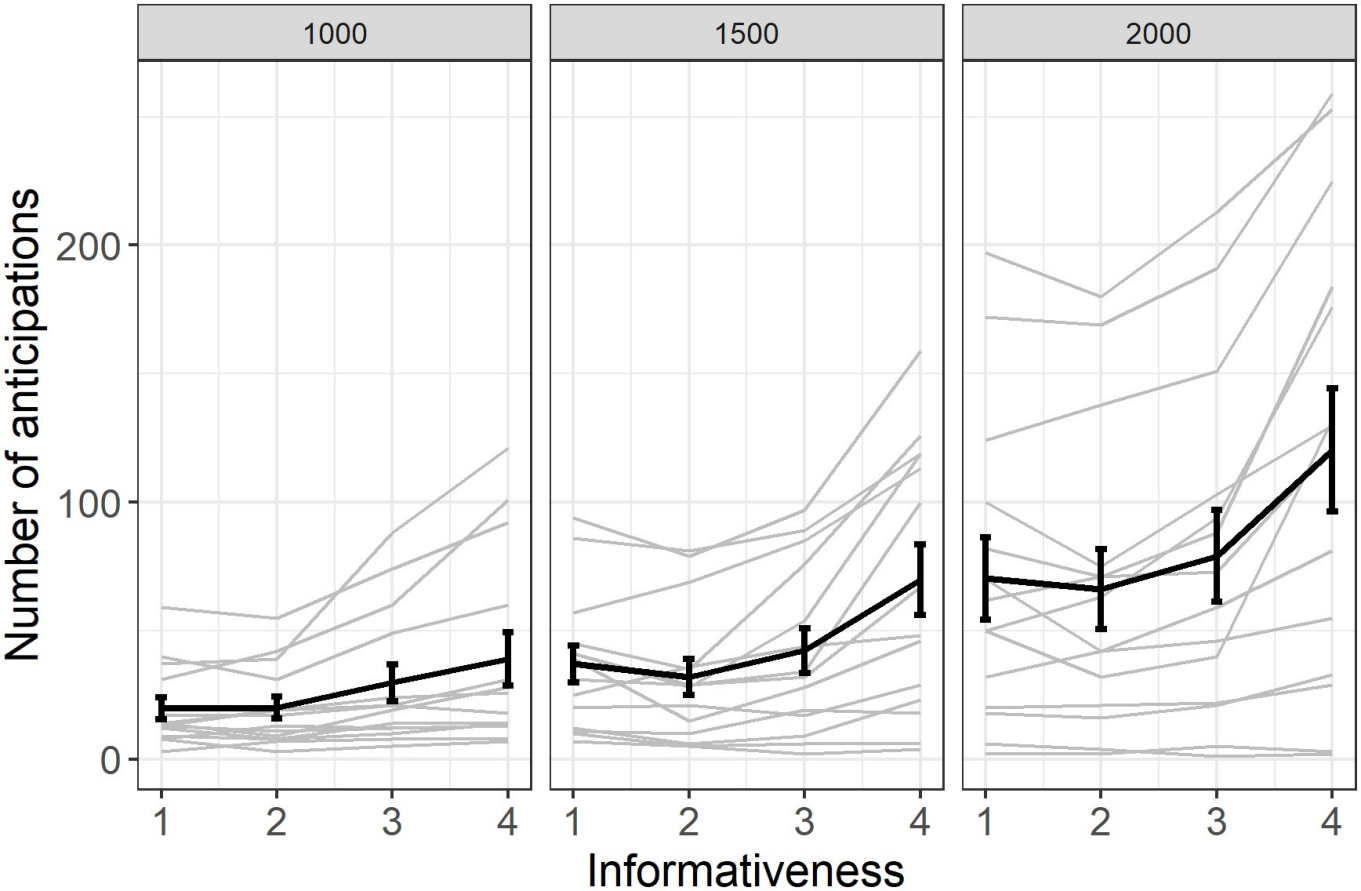
The number of anticipations increases with informativeness. Individual responses (light grey) with group average (error bars represent standard error).

As predicted, we found a strong and significant increase in the number of anticipations with informativeness (β = 0.20, SE = 0.02, z = 10.8, p < 0.001, see Fig 2), confirming that the monkeys used the informativeness of the cue to respond to the task (note that the effect of informativeness is similar in an analysis with the first three levels of informativeness only, β = 0.09, SE = 0.02, z = 3.7, p < 0.001).

**Fig 3:**
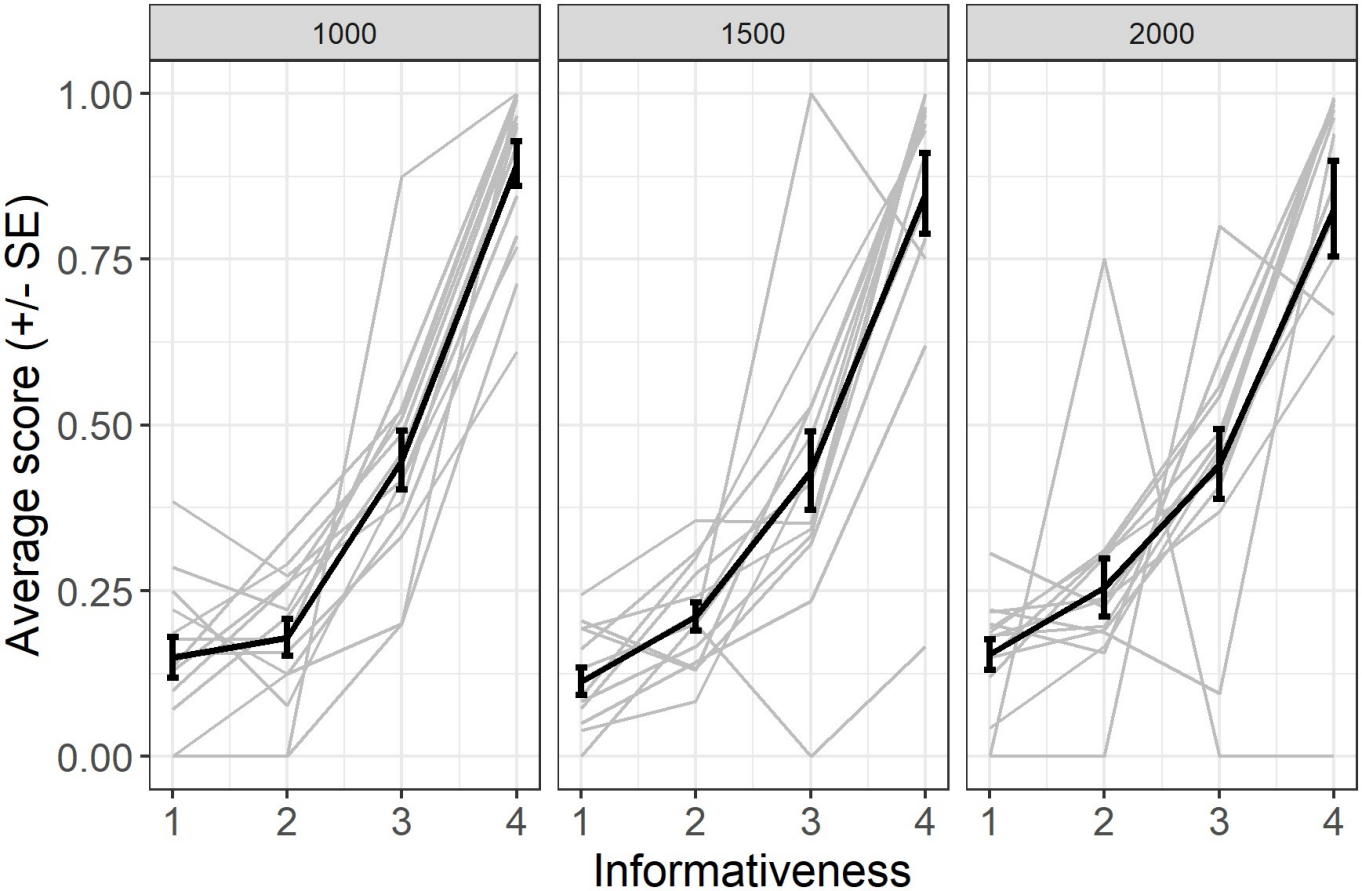
Average score (rewarded responses) for anticipated trials. Individual responses (light grey) with group average (error bars represent standard error).

As can be seen in Fig 3, the number of anticipations significantly increased with the delay (the model with delay was significantly better, Χ^2^ = 9.2, df = 2, p = 0.01) but this increase was uniform across levels of informativeness (the model with an interaction between delay and condition was not significantly better than the model without the interaction, Χ^2^ = 0.49, df = 2, p = 0.78).

Note that in the previous analyses we considered the total number of anticipations, including anticipations in which the cue and the image touched did not match. If we focus on ‘correct’ (reinforced) anticipations with a match between the cue and the stimuli touched, the effect of informativeness is even stronger (GLMM, β = 0.35, SE = 0.02, z = 18.2, p < 0.001; also see S2 Fig in S1 File) because the baboons tended to anticipate and touch the incorrect stimulus (a distractor) when it was presented on its own (i.e., when the level of informativeness is 1). This is certainly a consequence of the fact that the same configuration with a different cue would lead to immediate and certain reward (informativeness of 4).

We also explored if a relation exists between score and number of anticipations obtained in each condition of informativeness. Fig 3 reveals that the score increased with the level of informativeness of anticipation trials (GLMM, β = 1.46, SE = 0.03, z = 49.2, p < 0.001). This finding suggests that the baboons considered informativeness for selecting the most optimal response strategy in each trial and produced anticipation responses as a means to optimize reward delivery.

#### Decrease in reaction time for successful revealed trials

**Fig 4:**
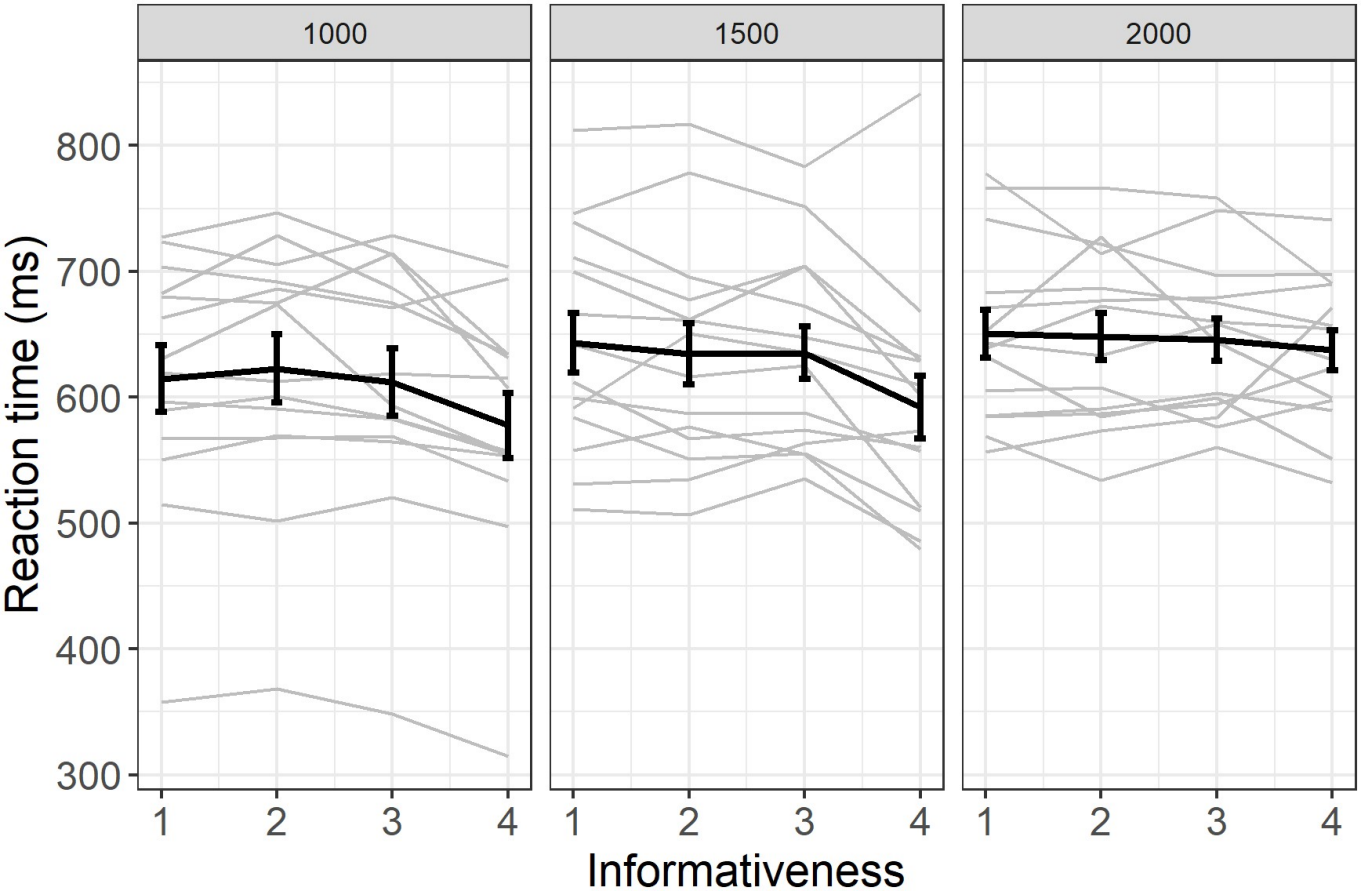
Change in reaction time with informativeness, when the target was revealed and the monkey was successful. The reaction time is the time it took the individual to respond after the target turned yellow (in millisecond). Individual responses (light grey) with group average (error bars represent standard error). Data from all the trials are represented in this figure but fast and slow responses were analyzed separately (see methods).

Fig 4 presents the effect of informativeness on reaction times for all successful revealed trials. We found a small and significant decrease in reaction time with informativeness for slower responses (LMM, β = -5.6, SE = 0.83, df = 40.8e+3, t = -6.6, p < 0.001), with no clear effect of delay.

**Fig 5:**
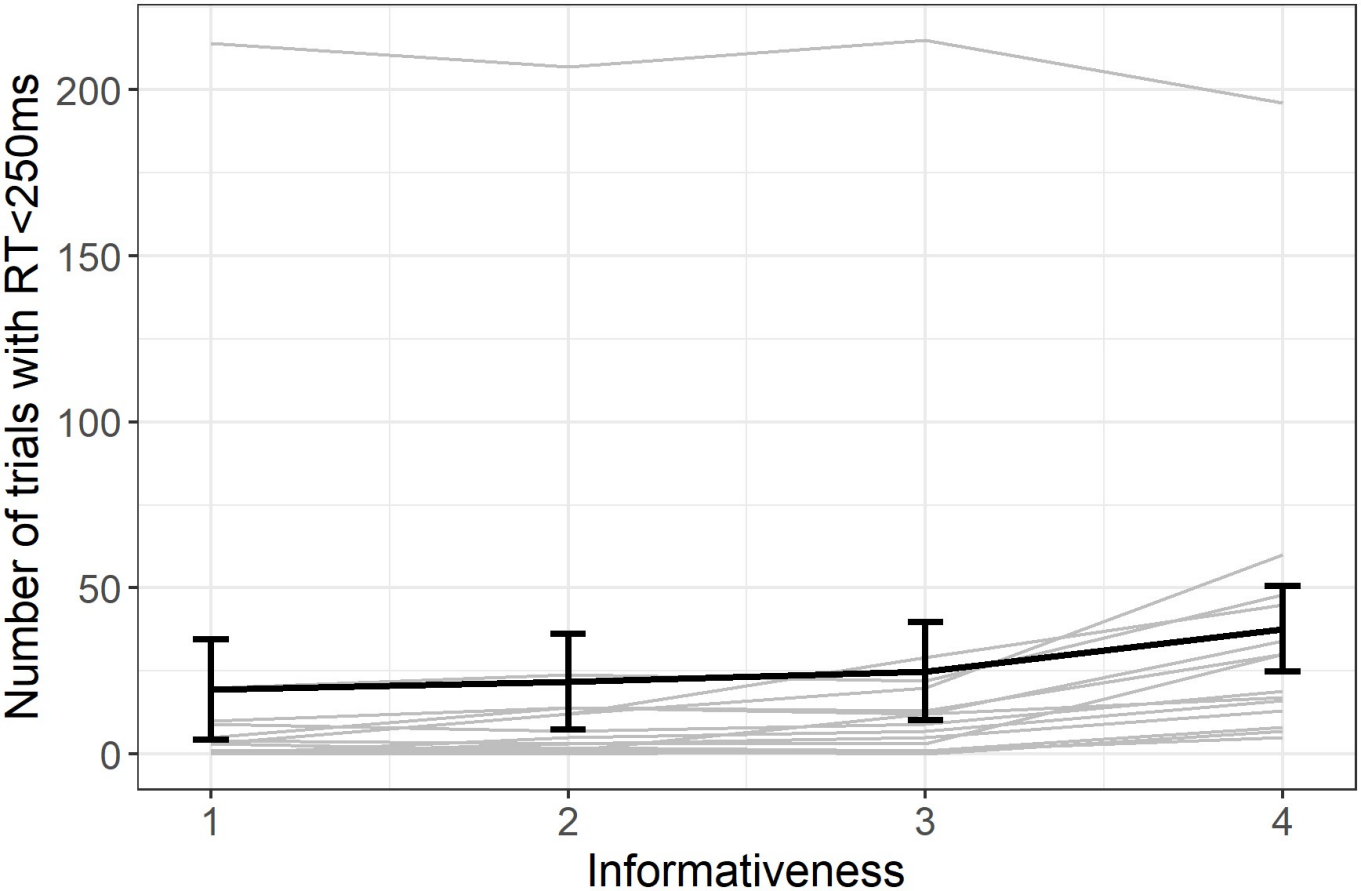
Number of fast responses (RT < 250ms) with informativeness. Individual responses (light grey) with group average (error bars represent standard error).

When analyzing fast responses (see Fig 5, we found an increase in the number of fast responses with informativeness (Spearman S = 14919, p = 0.001, ρ = 0.49). One monkey, ARIELLE, had many more fast responses than others. When excluding this individual, the correlation is preserved (S = 10049, p < 0.001, ρ = 0.57).

To summarize, Experiment 1 showed that baboons used informativeness through three different measures, an increased number of anticipations, decreased RTs for long responses and an increased number of fast responses. Importantly, the score also increased with informativeness on anticipated trials (Fig 3).

### Discussion

In Experiment 1, the informativeness of the cue was modulated by manipulating the number of stimuli similar to the cue in the response display (from 1 to 4). Importantly monkeys could respond immediately or wait for a delay (1000, 1500, 2000ms) until the target was revealed. All correct trials (i.e., choosing the S+) were rewarded regardless of whether they were anticipated or revealed. We had two hypotheses: anticipations should increase with informativeness; reaction times for revealed trials should decrease with informativeness. This is exactly what we found. Additionally, fast responses to revealed trials (< 250ms) increased with informativeness.

The results of Experiment 1 suggest that the baboons produced more anticipation responses and processed informativeness as a means to optimize food reinforcement. In Experiment 2, we wanted to check whether the responses in anticipated trials depended on informativeness alone, on reward tracking alone, or on a combination of both. To address that question, in Experiment 2 all anticipation responses in which the subject selected the same stimulus as the sample were evenly rewarded, independently of the level of informativeness of the display.

## Experiment 2

### Material and methods

#### Participants

Twenty Guinea baboons belonging to the same facility as in Experiment 1 participated in this study (median age 11 years, min = 3, max = 25; 5 males).

#### Training and test procedure

Subjects were retrained prior to the test sessions, using the same general training procedure as for Experiment 1, with the only exception that the response stimuli could be displayed in 6, rather than 5 possible locations on the screen. Testing was proposed after the baboons reached 80% correct for both the matching and revealed trials with an anticipation delay of 1000ms. Three test phases were then proposed to the baboons after retraining, using anticipation delays of 1000ms, 1500ms and 2000ms. The testing procedure was similar to Experiment 1 but involved four main changes. First, these test phases employed a “O” and a “T” shape as stimuli, rather than a circle and a square. Second, all response displays contained 6 shapes, instead of 5 as in Experiment 1. Third, the cue could be replicated 2, 3 or 4 times in the response display, resulting in only three levels of informativeness. Fourth and most importantly, all anticipations responses in which the subject selected the same response stimulus as the cue were non-differentially reinforced on a 70% rate, independently of the condition of informativeness. Incorrect anticipation responses were by contrast not reinforced. Responses occurring after the anticipation period were differentially reinforced with food rewards being delivered in the test cage after each correct response.

### Experiment 2: Results

Fourteen monkeys passed training and completed the full set of test trials (median age 11 years, min = 4, max = 23; 3 males).

#### Increase in number of anticipation trials

**Fig 6:**
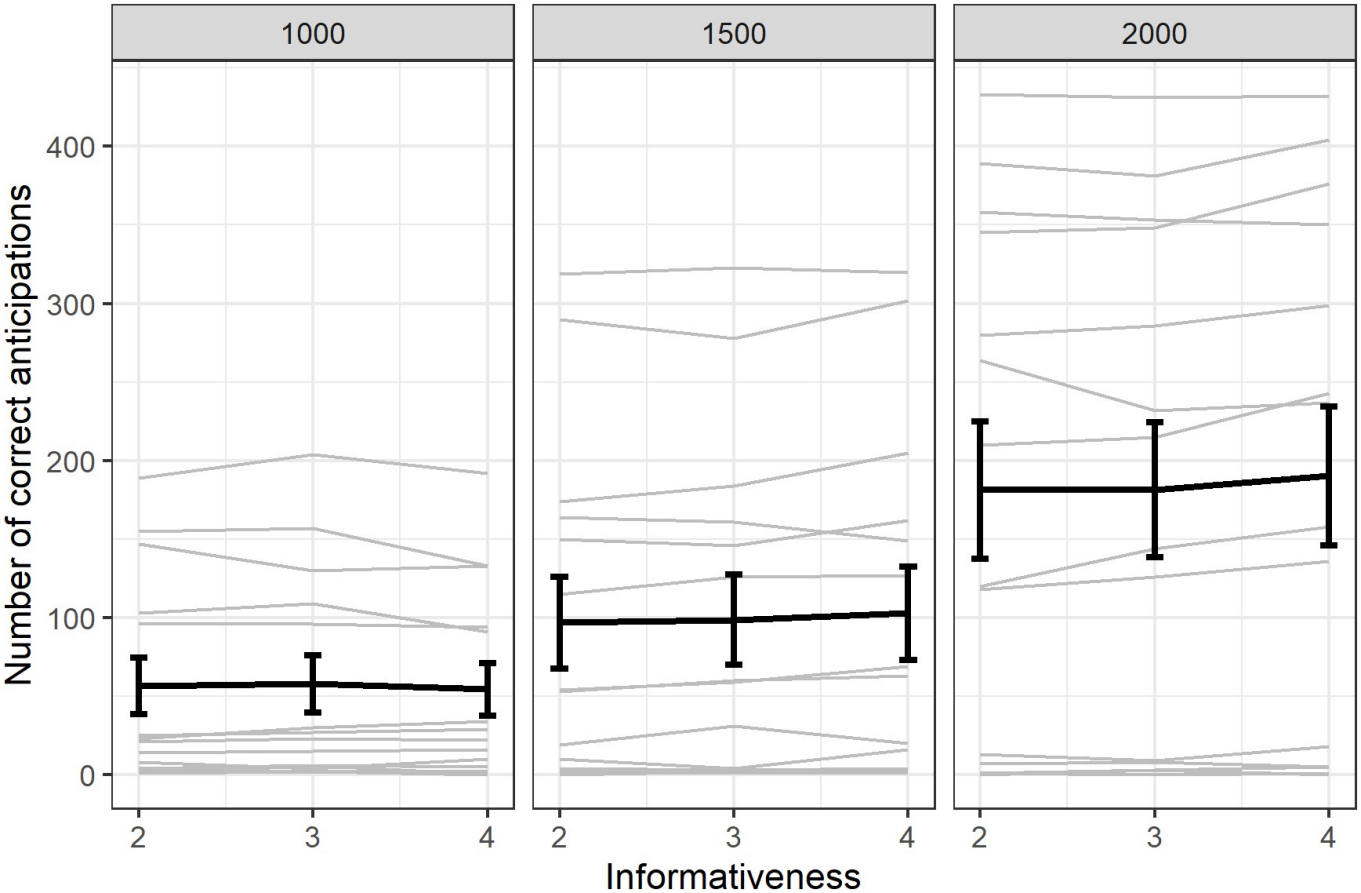
Number of anticipations depending on informativeness and delay. Individual responses (light grey) with group average (error bars represent standard error).

Compared to Experiment 1, we found no increase in the number of anticipations with informativeness (β = 0.04, SE = 0.08, z = 0.49, p = 0.63, Fig 6).

#### Decrease in reaction time for revealed trials

**Fig 7:**
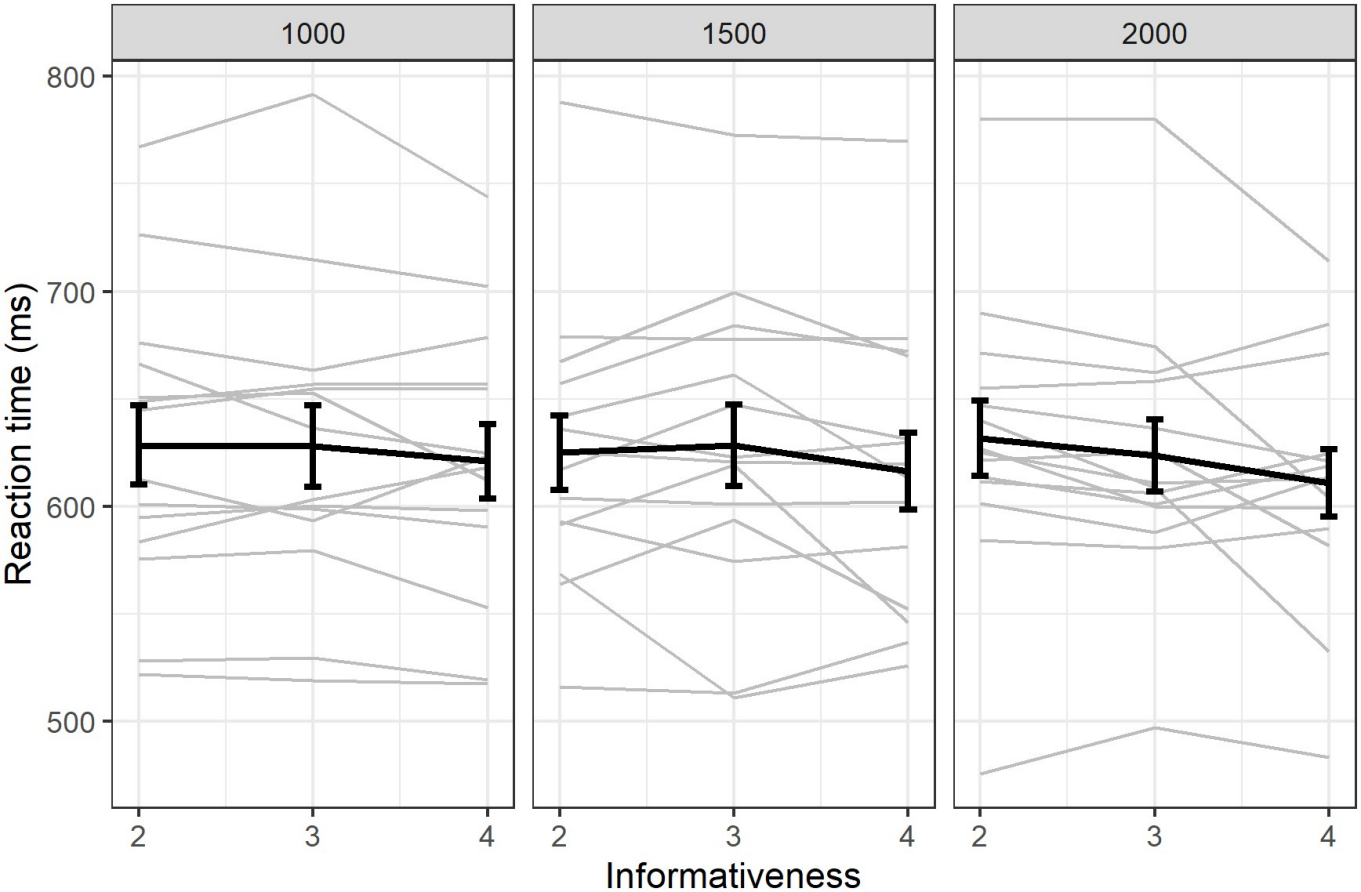
Reaction times for successful revealed trials. Individual responses (light grey) with group average (error bars represent standard error).

We also found a small decrease in reaction time with informativeness (β = -1.49, SE = 0.76, df = 39997, t = -1.95, p = 0.052; Fig 7).

#### Increase in the number of rapid responses in revealed trials

**Fig 8:**
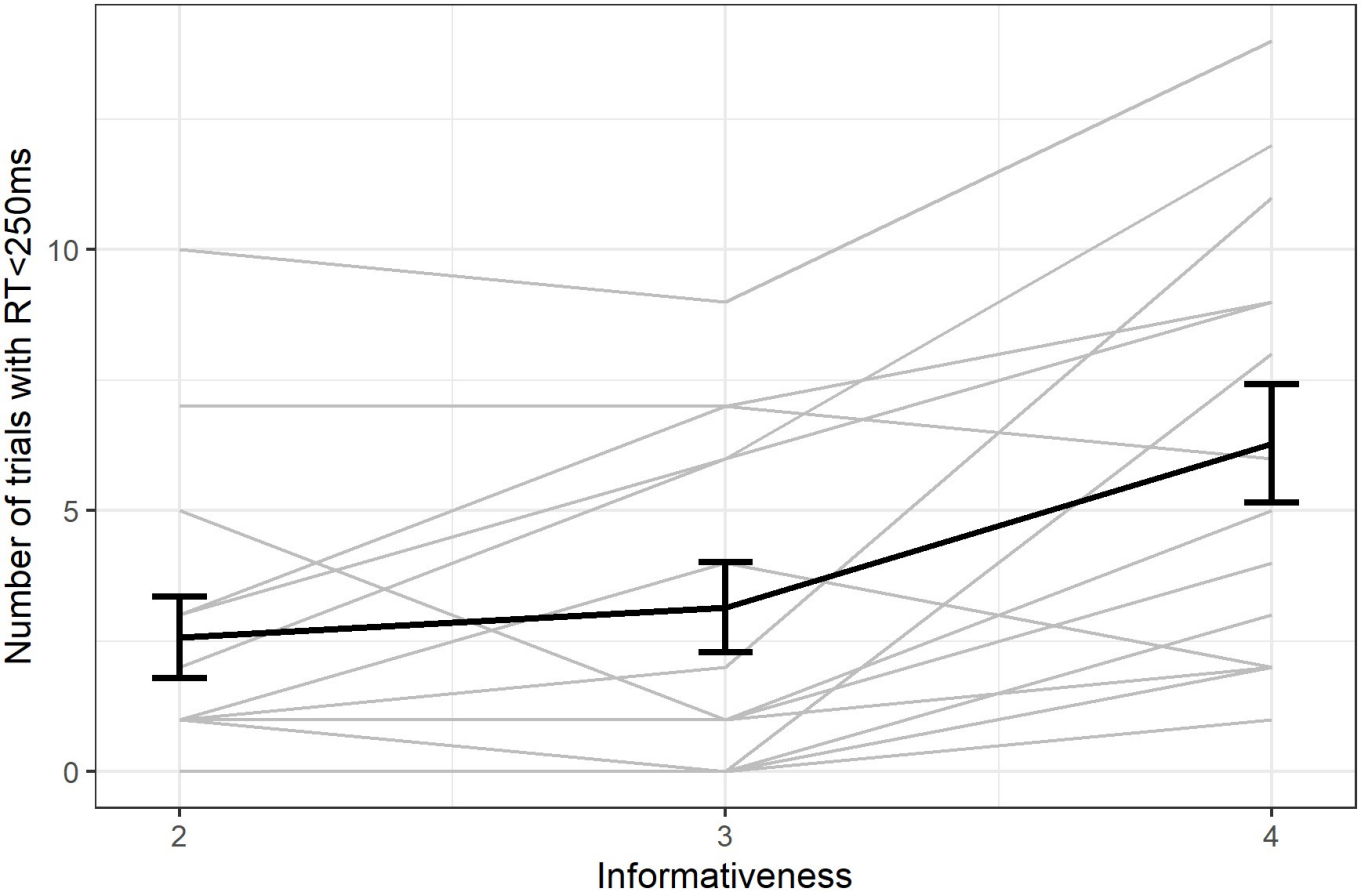
Number of correct revealed trials with RT<250ms. Individual responses (light grey) with group average (error bars represent standard error).

The number of successful trials with a rapid response (RT<250ms) increased with informativeness (Spearman rank, S = 7364, p-value = 0.008, ρ = 0.40; Fig 8).

### Discussion

Experiment 2 followed a similar procedure to Experiment 1, but importantly differed from the previous experiment regarding reinforcement contingencies of anticipation trials: In Experiment 2, anticipations were non differentially rewarded at 70% while correct revealed responses were rewarded at 100%. The main effect of this reward pattern was that, though anticipations increased with delay, they did not increase with informativeness, in contrast with the results in Experiment 1. On the other hand, there was still a decrease in reaction times for revealed trials with informativeness. There was also an increase in rapid correct revealed responses with informativeness. Thus, the main effect of the new reward pattern was to extinguish the effect of informativeness on anticipations, suggesting that the production of informativeness-dependent anticipation trials in Experiment 1 was controlled by a reward incentive to anticipate.

In Experiment 3, we wanted to see whether we could restore the effect of informativeness on anticipations. We reasoned that if the monkeys experienced one condition in which anticipations were always successful this might prompt them to anticipate more in the other conditions. Therefore, we decided to reintroduce the condition in which the response display contained a single repetition of the cue.

## Experiment 3

### Material and methods

#### Participants

The subjects were from the same group as above.

#### Training and test procedure

Baboons were all retrained prior to testing, using the same training procedure as in Experiment 2. Testing involved two new visual objects as stimulus (a reversed “L” shape and a pentagon) and 32 repetitions of a test block of 56 trials which was repeated in a random order. The test trials had 4 possible conditions of informativeness, corresponding to 1, 2, 3 or 4 repetitions of the cue within the response display of 6-items. The duration of the anticipation display was set to 2000ms in that case. All anticipation responses to the first condition of informativeness (one matching stimulus, informativeness = 5) were differentially reinforced with correct responses rewarded at 100%. Anticipation responses in the remaining conditions of informativeness were non-differentially reinforced on a 70% basis when the baboons touched a shape identical to the cue. By contrast, touching a distractor by anticipation was coded as an error and was not reinforced. Non anticipation responses, after the stimulus had turned yellow, were all differentially reinforced.

### Results

Fourteen Guinea baboons belonging passed the training phase in Experiment 3 (median age 10 years, min = 3, max = 23; 2 males).

#### Increase in number of anticipation trials

**Fig 9:**
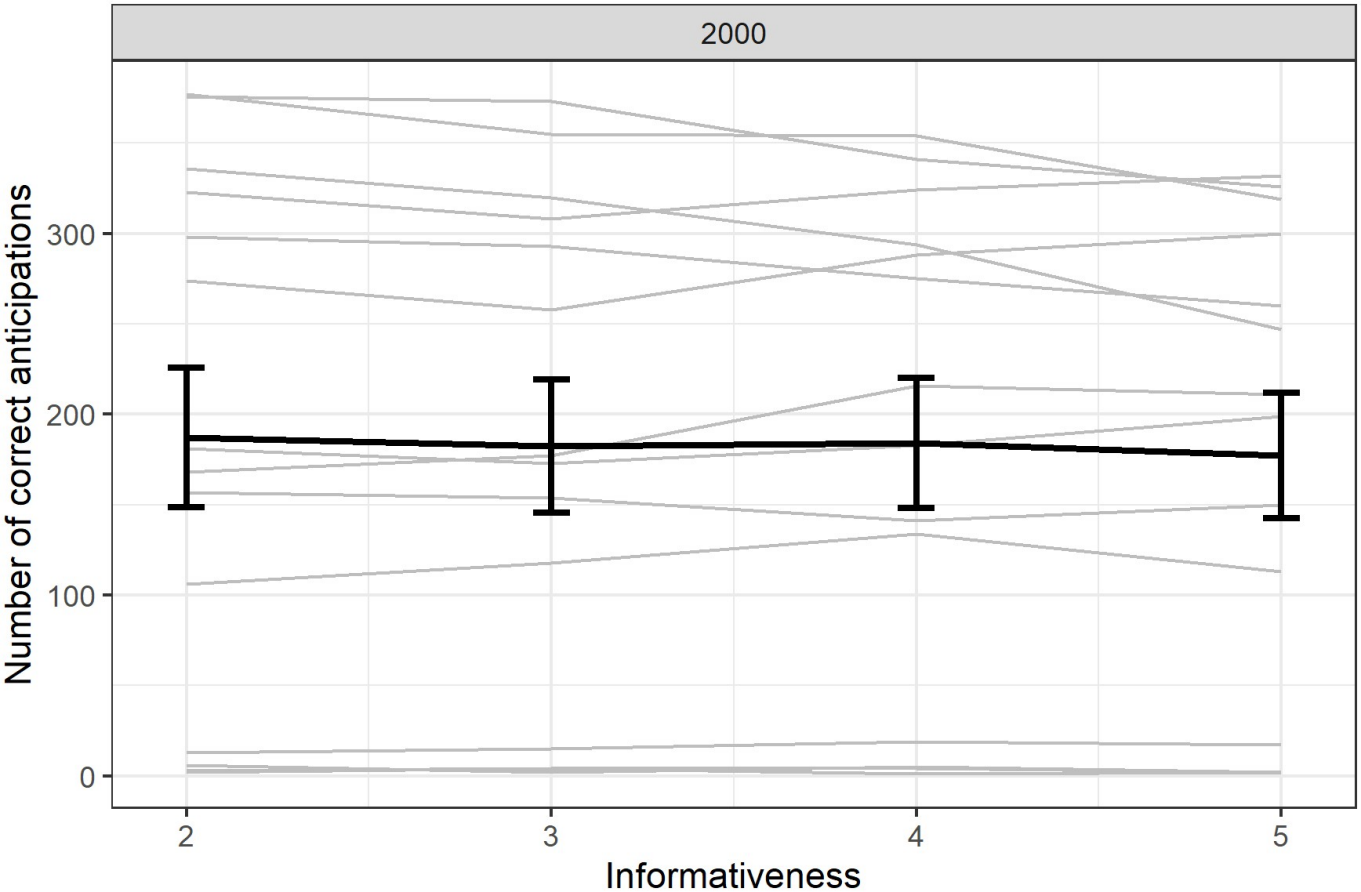
Effect of informativeness on the number of anticipations. Individual responses (light grey) with group average (error bars represent standard errors). In this experiment there was only one delay of 2000ms.

The same statistical analyses as above revealed no evidence of an effect of informativeness on the number of anticipations (β = -0.01, SE = 0.01, z = -1.15, p = 0.25; Fig 9).

#### Decrease in reaction time for revealed trials

**Fig 10:**
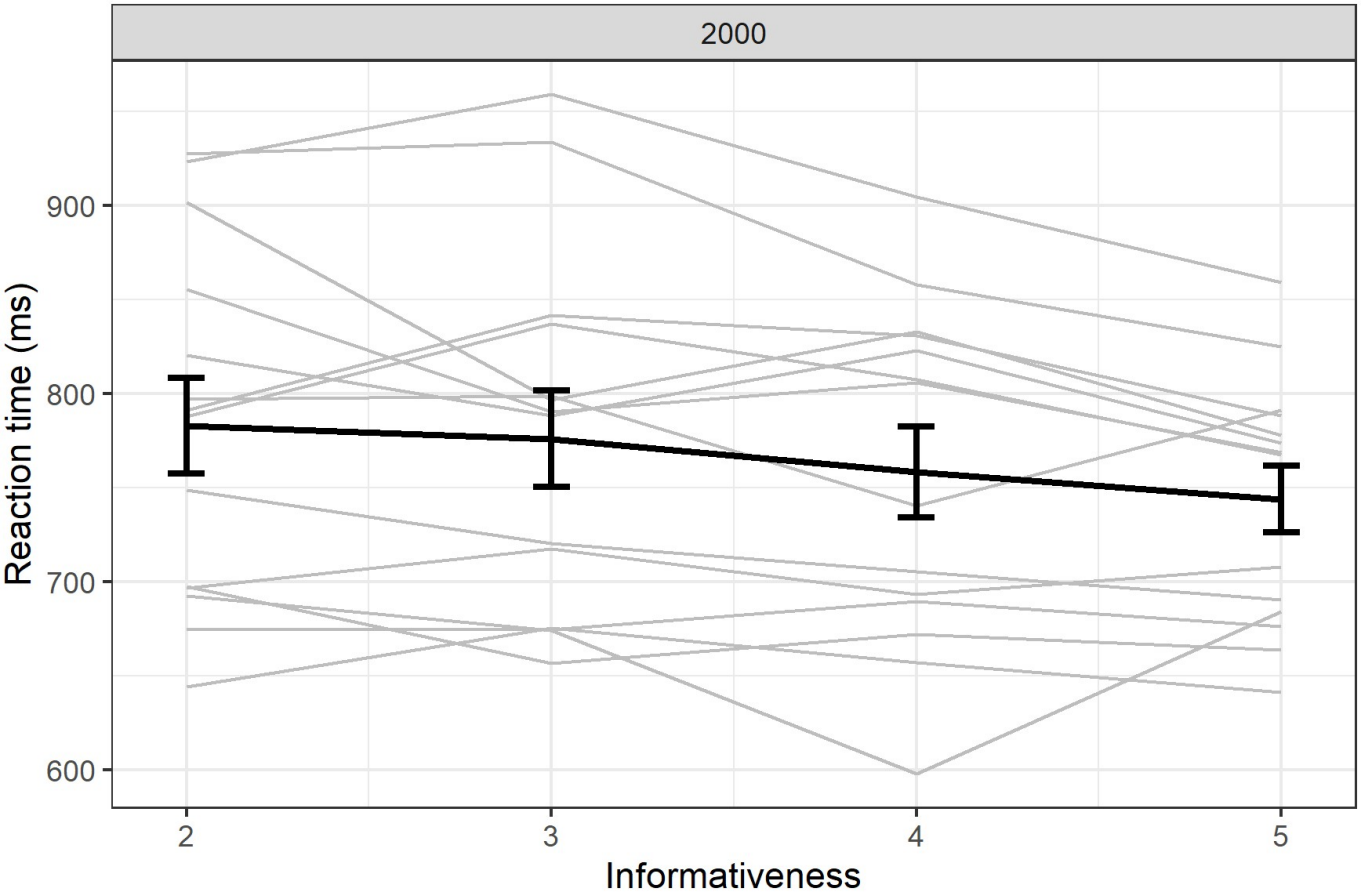
Reaction times for successful revealed trials. Individual responses (light grey) with group average (error bars represent standard error). In this experiment there was only one delay of 2000ms.

We also found a small decrease in reaction time with informativeness (β = -4.62, SE = 1.78, df = 13972, t = -2.60, p = 0.009; Fig 10).

#### Increase in the number of rapid responses in revealed trials

**Fig 11:**
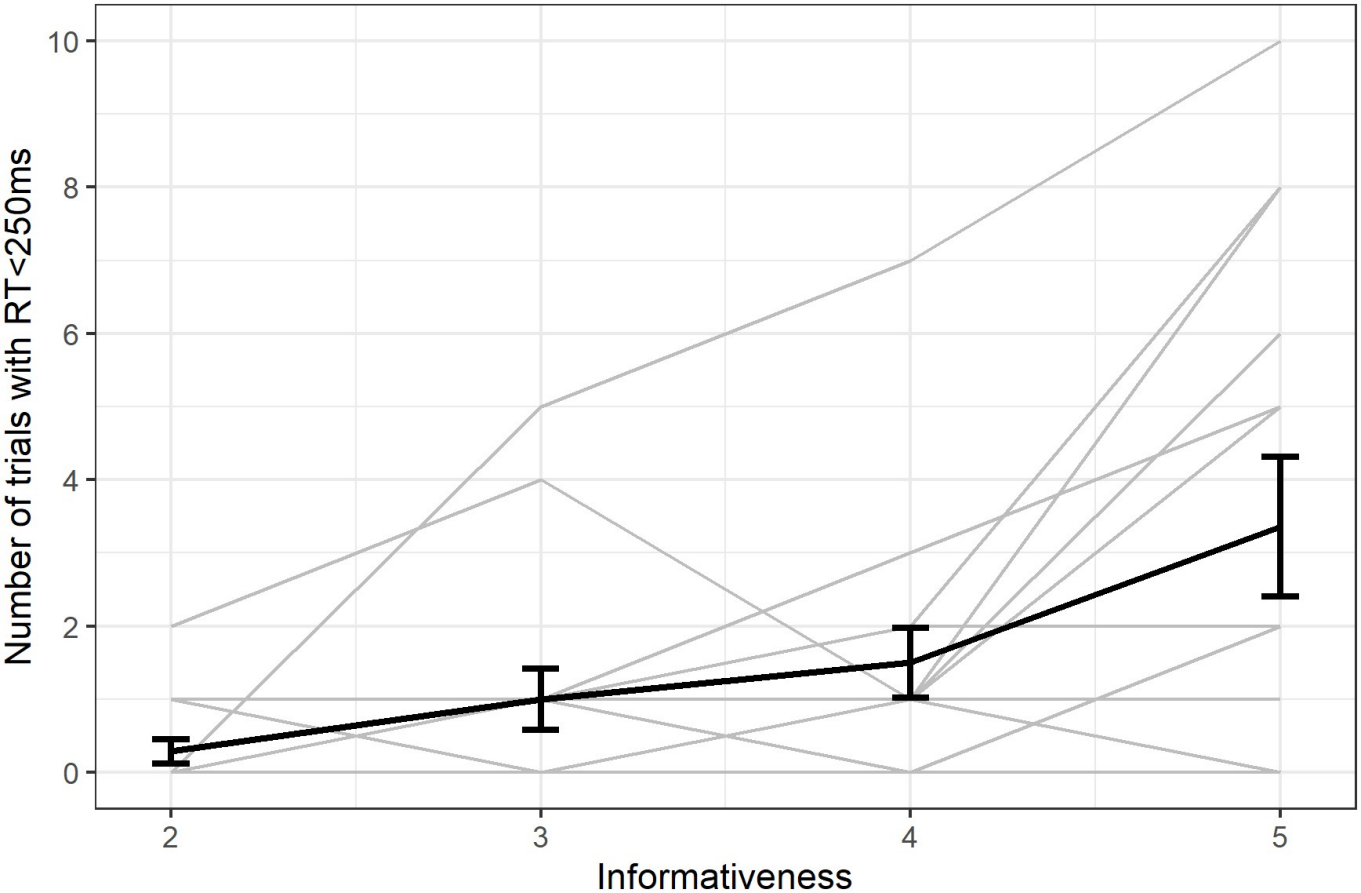
Increase in the number of correct revealed trials with RT<250ms. Individual responses (light grey) with group average (error bars represent standard error).

The number of successful trials with a rapid response (RT<250ms) increased with informativeness (Spearman rank, S = 6746, p-value = 0.002, ρ = 0.45; Fig 11, we excluded the condition with informativeness equal to five to make the comparison with the previous experiment valid).

### Discussion

Experiment 3 had three differences with Experiment 2, in addition to the use of new stimuli: (1) On the response display of 6 items, 1, 2, 3, or 4 items could be similar to the cue. (2) The delay was uniformly 2000ms, and (3) correct anticipations in the maximally informative condition (where a single item on the response display was similar to the cue) were differentially rewarded at 100%, while all other correct anticipations were only rewarded at 70%.

In Experiment 3, there was no effect of informativeness on the number of anticipations, as in Experiment 2 and in contrast with Experiment 1. On the other hand, there was a decrease in reaction times for correct revealed trials with informativeness. As well, there was an increase in correct fast responses to revealed trials with informativeness. Thus, the results of Experiment 3 were very similar to those of Experiment 2, despite the reintroduction of a maximally informative and differentially rewarded condition.

## General discussion

Our findings suggest several comments.

First, in experiment 1, the number of anticipations increased with the cue’s informativeness. Thus, the baboons waited less for additional evidence—provided here by the color change—when the cue was more informative. Importantly, in Experiment 1, the probability that an anticipation would be rewarded was positively correlated with the cue’s informativeness (Fig 4). Subsequently, in experiments 2–3, we equalized rewards for correct anticipations across several levels of informativeness. In these two experiments, the effect of informativeness on the participants’ number of anticipations disappeared completely. This set of results reveals that reward tracking might be one route supporting baboons’ capacity to adjust their behavior to a cue’s informativeness. Indeed, in experiments 2–3, we artificially dissociated reward patterns from the informativeness of the cue. However, in ecological contexts, informativeness and patterns of reward are intrinsically tied: Learning from cues that are more (rather than less) informative about the location of a reward makes one more likely to find it. Thus, tracking rewards (or successes) might allow baboons to perform a complex function—adjusting one’s behavior to the informativeness of cues—at a relatively low cognitive cost.

Second, the participants’ reaction times during the revealed trials decreased with the cue’s informativeness. Thus, the speed of baboons’ responses once the correct comparison stimulus (S+) changed color increased with the quality of evidence that they received during the anticipation period. Moreover, the number of fast responses (< 250ms post color change) increased with the cue’s informativeness. Given their speed, these fast responses were most probably prepared before the color change. These two findings are additional evidence supporting the idea that baboons have a sensitivity to informativeness. Importantly, the effects of informativeness on both reaction times and on the number of fast responses were observed in all three experiments, even after equalizing rewards for correct anticipations across levels of informativeness (Experiments 2–3). Thus, this second set of results reveals that baboons’ sensitivity to informativeness cannot be explained entirely by reward tracking, although reward tracking contributed to it.

Two types of cognitive mechanisms may explain the effect of informativeness on the speed of the participants’ responses. A first possibility is that baboons’ sensitivity to informativeness was underpinned by metacognitive abilities such as uncertainty monitoring. Under this rich interpretation, before the color change, the baboons represented the possible alternative locations of S+, along with an estimate of their own uncertainty over these alternatives. Understandably, more uncertainty about the reward’s location would result in slower responses from the participants.

A second—arguably leaner—account is that during the anticipation period, the participants adjusted their behaviors to the cue’s informativeness without monitoring their uncertainty. For instance, during the anticipation period, the participants might have prepared their response by dividing their attention over the repetitions of the cue. When the number of the cued shape’s repetition was larger, the participants needed to monitor more stimuli, thus slowing down their detection of the color change, and subsequently, their reaction times. Note that this interpretation still implies that during the anticipation period, the participants have a belief distribution about which stimuli in the response display could be the target. The wider the distribution of probability, the less certain the participant can be, though this uncertainty does not have to be represented as such. Such a mechanism would be computationally efficient, since it would allow baboons to slow down their responses when the evidence at their disposal was of lesser quality—yet it would not require that baboons monitor their uncertainty.

These results are strongly reminiscent of Hyman’s remark [30], based on Hick (1952) [31]: “When a stimulus is chosen to which S must make a discriminatory response, his reaction time seems to be a monotonically increasing function of the number of possible stimuli from which the stimulus can be chosen” (1953, 188). Hyman also notes that this type of experimental set-up “can be looked upon as a model of a communication system” (1953, 188). Indeed, the mechanisms evidenced here in baboons fulfill two desiderata that are crucial for many theories of contextually sensitive forms of communication (e.g., [2, 4]). First, they allow baboons to adjust their behaviors to the contextually dependent informativeness of cues. Indeed, in our study, there was nothing about the cued shape itself that made it intrinsically informative. Rather, the cue’s informativeness depended entirely on context, i.e., the distribution of shapes that the participants had to choose from. Second, as argued above, baboons’ sensitivity to informativeness may rely on relatively lean mechanisms that are nonetheless computationally efficient (e.g., [24]). From a more evolutionary standpoint, our data vindicate the view that a sensitivity to informativeness is already present in non-human primates, and shapes the way they use cues [7]. It can therefore be concluded that the cognitive ability to process informativeness pre-existed the emergence of language in the primate lineage.

## Supporting information

S1 File (DOCX)
