# Supplementary material for: Are monkeys sensitive to informativeness: An experimental study with baboons (Papio papio)
Source: PLoS One. 2022 Jul 5;17(7):e0270502. doi: 10.1371/journal.pone.0270502 (PMC9255764; doi:10.1371/journal.pone.0270502)
Supplement: S1 File — (DOCX) [file pone.0270502.s001.docx]

# Experiments

## Experiment 1


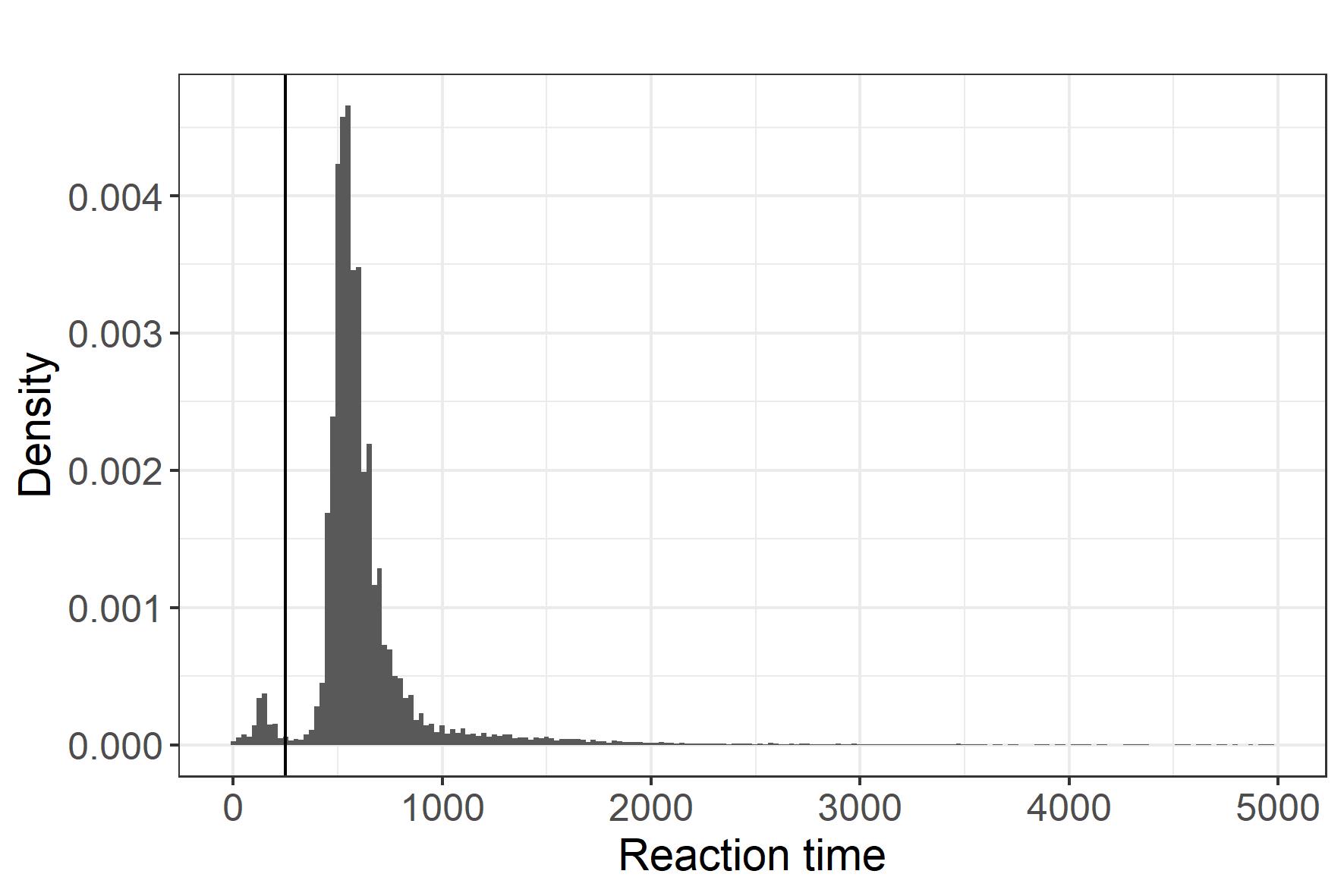


Figure S1: Bimodal distribution of reaction times for correct revealed trials. The figure shows two peaks in the distribution of reaction times, one around 200ms (fast responses) and one around 600ms (slow responses). The vertical line represents the 250ms cut off used in the analysis.


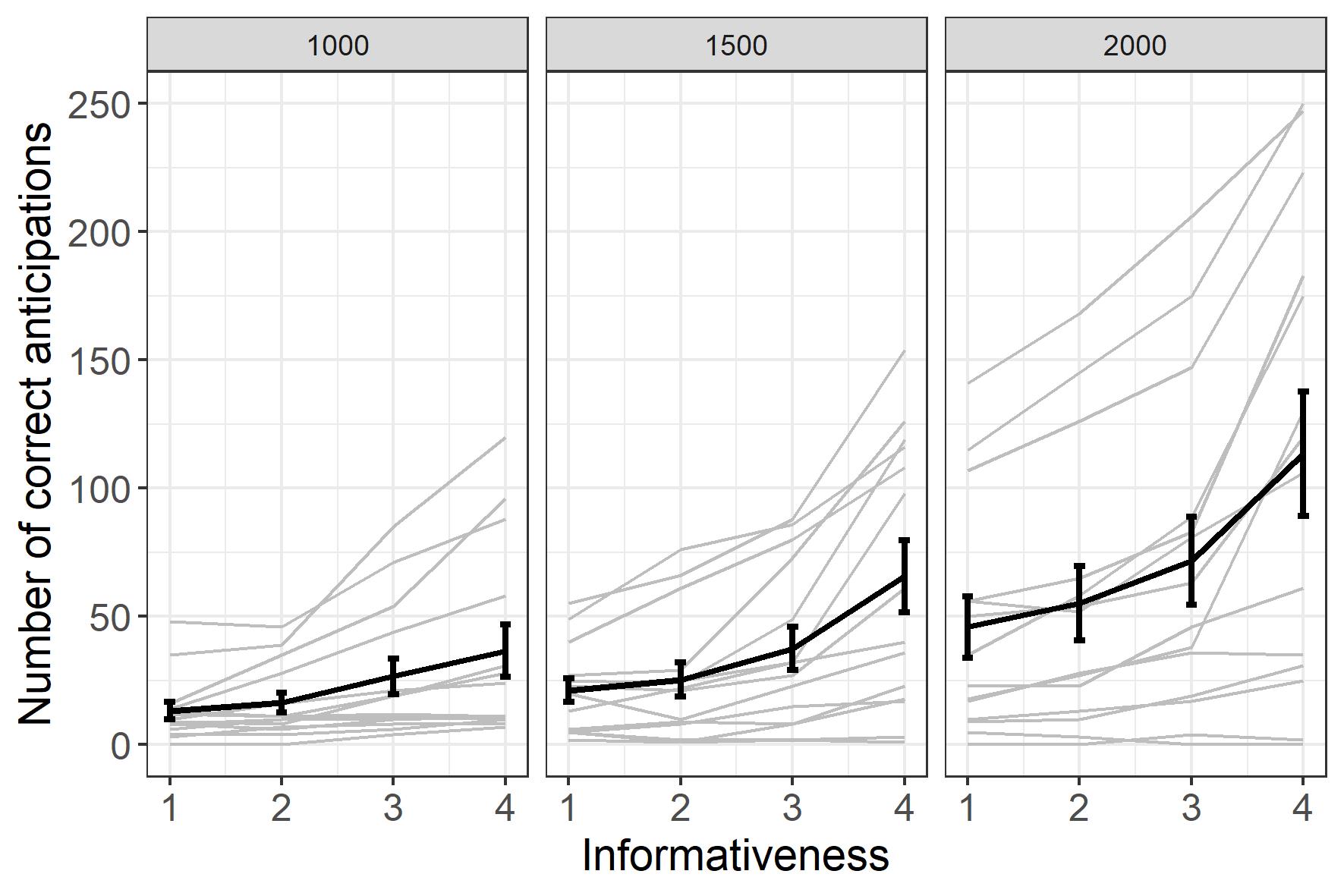


Figure S2: The number of ‘correct’ anticipations increases with informativeness. Correct anticipations are anticipation in which the cue and the stimuli touched are of the same category. Compared to figure 1 of the main text, we see that the effect of informativeness is stronger here, mostly because baboons make more incongruent choices when informativeness is equal to one (when the image touched is incorrect but is the only one present). Individual responses (light grey) with group average (error bars represent standard error).
